# Anterior uveitis and vasculitis in primary infection with VZV in a diabetic patient

**DOI:** 10.22336/rjo.2022.66

**Published:** 2022

**Authors:** Emma Marín Payá, Marina Aguilar González, Miriam Rahhal Ortuño, Paula Martínez López-Corell, María Andreu Fenoll, Patricia Udaondo Mirete

**Affiliations:** * Department of Ophthalmology, Hospital Universitari i Politecnic La Fe, Valencia, Spain

**Keywords:** oral corticosteroid, primary infection, retinal vasculitis, Type 1 diabetes mellitus, varicella-zoster virus

## Abstract

We present a case of ocular involvement in the context of a varicella zoster virus primary infection, in a 30-year-old man with type 1 diabetes. The ophthalmological examination revealed unilateral anterior uveitis resistant to topical treatment, which was complicated by association of vitritis and onset of retinal vasculitis diagnosed by angiography fluorescein. Herpetic eye involvement was confirmed by anterior chamber PCR for varicella- zoster virus. In addition, we started the treatment with oral valacyclovir 1 gram every 8 hours and 15 mg oral corticosteroid with very satisfactory results, without observing any side effects. Very few cases have been published on this topic, given the low prevalence of this ocular complication.

## Introduction

Varicella zoster virus (VZV) is a double-stranded DNA virus, from the Herpesviridae family. Primary infection is more common in children and develops with feverish disease and chickenpox dermatitis. During primary infection, viral particles migrate along the nerve endings until they reach the sensitive ganglion, where the virus is stationed. Subsequently, and due to different factors, such as the patient’s immunity, diabetes [**1**], stress, etc., the virus can be reactivated [**1**-**3**].

It is during the reactivation of VZV that herpetic ocular involvement usually occurs, with a small percentage of patients with ocular involvement during herpetic primary infection [**4**,**5**].

The most frequent ocular symptoms during the primary infection with VZV would be eyelid blisters, conjunctivitis, superficial punctate keratitis and possibly some atypical dendritic lesions. 

Anterior uveitis and retinal involvement are more frequent during the reactivations of the disease [**6**-**9**].

The article presents an unusual ophthalmological case during primary VZV infection in a 34-year-old diabetic adult, two weeks after the disappearance of varicelliform skin lesions. The symptoms began with anterior uveitis resistant to corticosteroid topical treatment, also complicated with vitritis and retinal vasculitis.

To date, there are very few published cases of retinal vasculitis due to primary infection of VZV, and even less with an association of anterior uveitis [**8**].

## Case report

We present the case of a 34-year-old type 1 diabetic patient who attended the Ophthalmology Emergency Department for loss of vision in his right eye for 4-5 days. Two weeks before, he was diagnosed with chickenpox and treated with 25 mg hydroxyzine (Atarax®), at 24 hours, for 7 days.

Ophthalmological examination revealed good pupillary reaction and the best corrected visual acuity was 0.9 in his right eye (RE) and 1 in his left eye (LE). Intraocular pressure was 25 mmHg in his RE and 16 mmHg in his LE. On scanning under electronic biomicroscope, the RE showed a clear cornea with a slight tyndall ++, no iris atrophy, no keratic precipitates and no infiltrates. Moreover, no pathological findings were found in his LE. 

The patient was diagnosed with unilateral anterior uveitis in the right eye. The treatment with topical corticosteroid descending regimen, topical beta-blockers every 12 hours, and cycloplegic eye drops every 8 hours was started.

At one-week follow-up, the scan was unchanged, so the treatment was maintained, but two weeks after the start of uveitic treatment, an increase of inflammatory cellularity in the anterior chamber of the RE (tyndall +++) and lower corneal endothelial deposits with normal ocular pressure (in treatment with topical beta-blocker) were observed. Fundus examination showed vitritis and an apparent bloodless vessel.

**Fig. 1:**
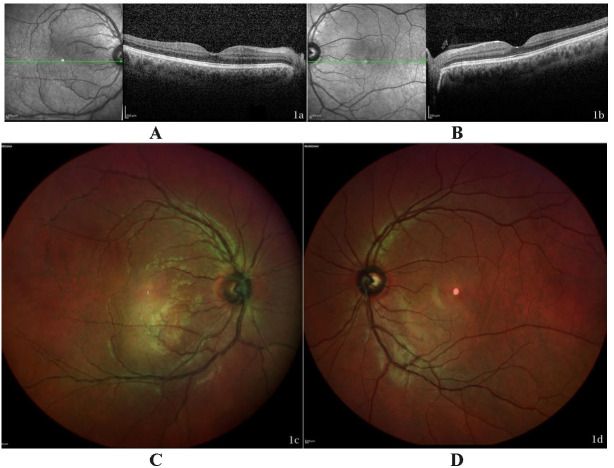
OCT SD and Multicolor of both eyes. Vitritis is seen in the right eye OCT **(A)**. Apparent vascular involvement in Multicolor of his right eye **(C)**. No pathological findings were found in his LE **(B, D)**

**Fig. 2:**
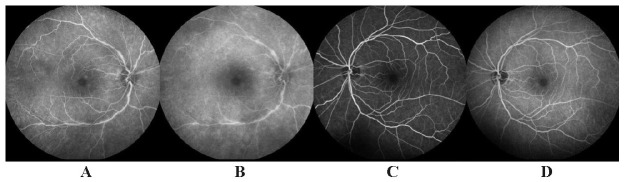
Fluorescein angiography showed vasculitis in the late phase in the right eye **(A, B)** and normal findings in the left eye **(C, D)**

Given these findings, we performed an optical coherence tomography (OCT) (**Fig. 1 A, B**), Multicolor image (**Fig. 1 C, D**), and a fluorescein angiography (FA) in early and late phase (**Fig. 2**).

In the late phase FA of the right eye, we could visualize the onset of temporal arcade vasculitis (**Fig. 2 B**).

We took a sample of aqueous humor (HA) in the right eye requesting PCR for VZV.

We added 15 mg of oral glucocorticoid and valaciclovir 1 gram every 8 hours to the initial treatment for anterior uveitis.

One week later, the results of the aqueous humor sample were positive for VZV DNA. We continued Valaciclovir 1 gram every 8 hours until completing a month. 15 mg of glucocorticoid were tapered down every 10 days. Topical corticosteroids treatment was continued weekly together with a descending regimen, topical beta-blocker and mydriatic.

**Fig. 3:**
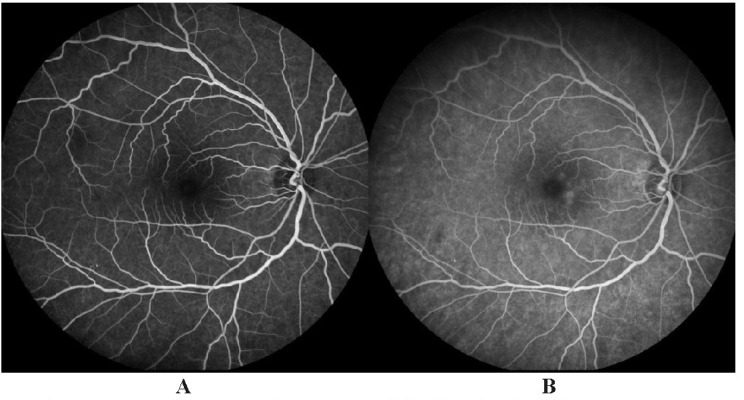
Fluorescein angiography showed normal findings in the right eye one month after treatment

One month after starting the treatment, multimodal imaging was carried out and vitritis was no longer observed. Right eye fluorescein was normal (**Fig. 3**).

We continued treatment with valaciclovir 500 mg every 12 hours and glucocorticoid 2.5 mg/ day and topical corticosteroids for another month, until the descending regimen was finished.

Currently, after 3 months of follow-up, the patient is asymptomatic, without any treatment and is free of ocular involvement.

## Discussion

Reviewing the literature, we have seen that both anterior uveitis and retinal vasculitis are rare in the context of VZV primary infection [**8**,**9**].

Initial treatment for anterior uveitis during VZV primary infection usually involves topical corticosteroids and oral antivirals, as performed by Kuo YH and col. or Wong F et al. [**8**,**10**]. However, the initial treatment for vasculitis in the context of primary VZV infection is not protocolized given the low prevalence of said involvement. There are few published cases, from which we took into consideration the case of Kuo YH as a reference, given the great similarity of the patient - the case of a 30-year-old woman who, after two weeks of the disappearance of the skin lesions, developed VZV vasculitis. In this case, treating only with oral antivirals (valacyclovir) has solved the vasculitis complication [**8**].

The difference in our patient’s case is that he was a diabetic with anterior uveitis resistant to topical corticosteroid treatment.

In a young diabetic patient with uveitis and vasculitis secondary to VZV, we decided to start valaciclovir 1 gram every 8 hours and low-dose corticosteroids, starting treatment with 15 mg. The reason for adding the oral corticosteroid, not described in these cases except for resistance to antiviral treatment, was the fast evolution, given the immunosuppressive state of the DM1 patient, as well as the lack of response of the anterior uveitis to topical treatment, trying to stop the inflammation of both the posterior pole and the anterior pole in a more radical way.

After this treatment, we were able to verify the excellent result by stopping vasculitis and vitritis in less than a week, as well as stopping the previous uveitis that did not initially respond to topical treatment. We did not find any sequel to the use of oral corticosteroids in this patient.

Antiviral treatment was maintained for 2 months, and oral corticosteroid treatment was maintained for two months in a slow descending pattern. Currently, the patient is with AV unit and free of ocular involvement.

## Conclusion

The results have been successful, so we consider that adding oral glucocorticoids to the usual antiviral treatment could help stop complications more effectively within clinical safety in these rare viral complications.

**Conflict of Interest statement**

The author(s) declare no potential conflicts of interest with respect to the research, authorship and/ or publication of this article. 

**Informed Consent and Human and Animal Rights statement**

The patient(s) consented to the publication of this case and the use of the images. 

**Authorization for the use of human subjects**

Ethical approval: The research related to human use complies with all the relevant national regulations, institutional policies, is in accordance with the tenets of the Helsinki Declaration, and has been approved by the review board of Hospital Universitari i Politecnic La Fe, Valencia, Spain.

**Acknowledgements**

None.

**Sources of Funding**

The author(s) received no financial support for the research, authorship and/ or publication of this article.

**Disclosures**

None.
